# Association between oxidative balance score with cardiovascular - kidney - metabolic syndrome and all-cause mortality among US population

**DOI:** 10.3389/fnut.2025.1564368

**Published:** 2025-08-05

**Authors:** Lin Shi, Dan Zhang, Shuangshuang Zhang

**Affiliations:** ^1^Department of Gastroenterology, Xuzhou Central Hospital, Xuzhou Clinical School of Xuzhou Medical University, Xuzhou, China; ^2^Hepatopancreatobiliary Center, Beijing Tsinghua Changgung Hospital, Beijing, China; ^3^Department of Nephrology, Affiliated Hospital of Xuzhou Medical University, Xuzhou, China

**Keywords:** cardiovascular - kidney - metabolic syndrome, oxidative balance score, mortality, cohort study, data

## Abstract

**Background:**

Cardiovascular-Kidney-Metabolic (CKM) syndrome is a multi-stage condition with significant public health implications. The oxidative balance score (OBS), which integrates dietary and lifestyle pro-oxidants and antioxidants, offers a novel approach for evaluating oxidative stress in relation to CKM stages and outcomes. This study aimed to investigate the association between OBS and advanced CKM stages, as well as its relationship with all-cause mortality in a US population.

**Methods:**

Data were derived from the National Health and Nutrition Examination Survey. CKM stages were classified based on published criteria, and OBS was calculated using 20 components reflecting oxidative stress balance. Logistic regression and Cox proportional hazards models were applied to evaluate the associations between OBS and advanced CKM stages, and all-cause mortality, respectively. Restricted cubic spline (RCS) analyses were used to assess the potential nonlinear relationships.

**Results:**

A total of 12,793 participants aged 30–79 years were enrolled. A higher OBS was inversely associated with advanced CKM stages (adjusted OR for T3:0.58, 95% CI 0.46–0.73). A significant inverse relationship was also observed between OBS and all-cause mortality across the CKM stages. In non-advanced CKM stages, the adjusted HR for T3 was 0.58 (95% CI 0.40–0.86). In advanced CKM stages, the inverse association persisted (adjusted HR for T3: 0.62, 95% CI 0.43–0.89). RCS analyses confirmed a dose–response relationship between higher OBS and reduced mortality risk.

**Conclusion:**

This study highlights the protective role of higher OBS against advanced CKM stages and mortality, suggesting the potential of oxidative stress modulation as a strategy for managing CKM syndrome.

## 1 Introduction

Cardiovascular-Kidney-Metabolic (CKM) syndrome, as defined by the American Heart Association, is a multi-stage, multi-system condition encompassing the interplay between obesity, diabetes, chronic kidney disease (CKD), and cardiovascular disease (CVD) (1). Epidemiological studies indicate an alarmingly high prevalence of CKM syndrome among US adults, with approximately 90% meeting the criteria for CKM stage 1 or higher, and 15% classified in advanced CKM stages (3 or 4) (2, 3). This syndrome poses a substantial public health burden, associated with premature mortality, increased morbidity, and multi-organ disease, leading to escalated healthcare expenditures, particularly among vulnerable populations (4, 5).

CKM syndrome exhibits a progressive nature, typically originating from biological, social, and environmental exposures or stressors in early life. These factors lead to excessive and dysfunctional adipose tissue accumulation, subsequently promoting the development of metabolic risk factors (such as hypertension, hypertriglyceridemia, and diabetes) and CKD (4, 6). Non-advanced CKM stages (0–2) range from normal health to moderate metabolic or kidney risk, while advanced stages (3 or 4) involve severe kidney dysfunction or established cardiovascular disease. The staging model of CKM syndrome emphasizes the importance of early identification of CKM-related changes to support preventive efforts and highlights the stepwise increase in absolute CVD risk as the disease progresses, with intensive treatment in later stages yielding the greatest clinical benefits. Focusing on these stages is crucial to elucidate drivers of progression and guide targeted interventions for at-risk populations.

Oxidative stress, characterized by an imbalance between oxidative and antioxidant systems leading to excessive reactive oxygen species (ROS) accumulation, plays a central role in CKM syndrome pathophysiology (5). In cardiovascular disease, oxidative stress promotes atherosclerosis, endothelial dysfunction, and vascular inflammation (7–10). It activates angiotensin II and vasoconstrictive factors, leading to hypertension and cardiac remodeling (9, 10). In kidney disease, oxidative stress contributes to declining glomerular filtration rates and tubulointerstitial injury, particularly in diabetic nephropathy (11, 12). Moreover, oxidative stress is closely linked to insulin resistance, a core feature of metabolic syndrome and type 2 diabetes, promoting inflammatory responses in adipose tissue and the development of metabolic risk factors (13).

The complex interplay between oxidative stress and diseases has led to increasing interest in comprehensive assessment methods. The oxidative balance score (OBS), which incorporates both pro-oxidant and antioxidant factors from diet and lifestyle, has emerged as a promising tool (14). OBS components typically include fifteen antioxidants (such as vitamins C, E, β-carotene, and dietary fiber) and five pro-oxidants (including total fat, iron, alcohol intake, body mass index, and cigarette use) (14). Previous studies have demonstrated associations between OBS and various chronic diseases, including type 2 diabetes, cardiovascular disease, and certain cancers (15–18). Higher OBS has been linked to reduced all-cause mortality and cardiovascular disease-related mortality, emphasizing the importance of antioxidant-rich diets and healthy lifestyle practices in chronic disease prevention (11, 19).

Despite these emerging findings on the relationship between OBS and various chronic diseases, there remains a gap in understanding the specific association between OBS and CKM syndrome severity, particularly in distinguishing between advanced and non-advanced CKM stages. Therefore, this study aims to investigate the association between OBS and advanced versus non-advanced CKM stages, as well as its relationship with all-cause mortality. Understanding these relationships could help identify modifiable factors for intervention and guide the development of more effective, personalized approaches to managing CKM syndrome, particularly for those at risk of or already in advanced stages.

## 2 Methods

### 2.1 Study population

This cross-sectional study used data from the National Health and Nutrition Examination Survey (NHANES), a serial, cross-sectional, national survey with a complex, stratified, multistage probability design. Conducted by the U. S. Centers for Disease Control and Prevention, NHANES assesses the health and nutritional status of the noninstitutionalized U. S. population, providing nationally representative data on risk factors, biomarkers, and disease prevalence. In our analysis, we drew upon data from four discrete cycles of the NHANES, conducted between 2009 and 2016. The exclusion criteria were: 1. Participants with incomplete data to assess CKM stages. 2. Individuals lacking sufficient data to calculate OBS. 3. Participants with incomplete covariate data. Additionally, we omitted participants for whom the 10-year CVD risk could not be evaluated using the American Heart Association’s PREVENT equations, which are crucial for defining CKM stages and are applicable to individuals aged 30 to 79 years (3, 20, 21). As a result, the dataset for our analysis was confined to individuals within the age range of 30 to 79 years. Extensive information about the NHANES methodology and data collection procedures can be found in previous publications. The process began with a home interview to gather demographic information, followed by an examination at a mobile examination center to collect additional data (22, 23).

From the NHANES study spanning 2009–2016, a total of 40,439 participants were initially assessed. After applying the exclusions for incomplete data necessary for calculating the 10-year CVD risk or assessing CKM stages, the number of participants was reduced to 15,300. The final analysis encompassed 12,793 participants who had both complete data and complete OBS, after further excluding an additional 2,507 individuals (as depicted in Supplementary Figure 1).

### 2.2 Diagnosis of CKM syndrome

CKM syndrome was diagnosed based on the studies published by Aggarwal et al. (2), Zhu et al. (3), and Tang et al. (20). The syndrome is classified into five stages (0 to 4), with stages 3 and 4 designated as the advanced stages of CKM syndrome. The diagnostic criteria for each stage are shown in Supplementary Table 1. The 10-year CVD risk assessment is conducted using the PREVENT equation. A high risk of CVD was delineated by a 10-year risk threshold of no less than 20%. The risk of CKD and the calculation of estimated glomerular filtration rate (eGFR) followed the KDIGO guidelines.

To summarize, participants were categorized into five distinct CKM stages based on specific criteria: Stage 0: No abnormalities were observed. Stage 1: Characterized by obesity or prediabetes alone. Stage 2: Involved individuals with at least one additional metabolic disorder or moderate to high risk of CKD according to KDIGO guidelines. Stage 3: Defined by the presence of high 10-year CVD risk along with metabolic disorders or very high risk of CKD. Stage 4: A history of cardiovascular disease (e.g., coronary artery disease, angina, myocardial infarction, heart failure, or stroke). The detailed diagnostic criteria for each stage are shown in Supplementary Table 1.

### 2.3 Calculation of oxidative balance score

The oxidative balance score was calculated to assess the balance between pro-oxidant and antioxidant factors in participants’ diets and lifestyles (16, 18, 24). The OBS model incorporated a total of 20 components, classified into 5 pro-oxidants and 15 antioxidants. Pro-oxidants included total fat, iron, serum cotinine, alcohol consumption, and body mass index (BMI). antioxidants encompassed dietary fiber, β-carotene, vitamins (B2, niacin, B6, total folate, B12, C, E), minerals (calcium, magnesium, zinc, copper, selenium), and physical activity. Dietary nutrient and alcohol intake were assessed based on the average of two 24-h dietary recalls, conducted in person at a mobile screening center and subsequently by telephone within 3 to 10 days. These recalls included both weekdays and weekend days. Nutrient intake was estimated using the Food and Nutrient Database for Dietary Studies (FNDDS), developed by the U. S. Department of Agriculture. To ensure accuracy, dietary supplements and medication intake were excluded from the analysis. Physical activity was quantified using metabolic equivalents (METs) derived from questionnaire data, calculated as the product of metabolic equivalent score, frequency of each physical activity per week, and duration of each physical activity. Serum cotinine was utilized as a biomarker for both active and passive smoking, providing an accurate reflection of individual smoking levels. The overall OBS was calculated as the sum of scores for each component. Antioxidant components were assigned scores of 0, 1, and 2 from the lowest to highest tertiles, whereas pro-oxidants were scored inversely, with higher scores indicating higher antioxidant levels and lower scores indicating higher pro-oxidant levels (17). This comprehensive approach to OBS calculation allows for a nuanced assessment of the oxidative stress status within the study population.

### 2.4 Other variables

This research utilized the NHANES database to conduct a comprehensive analysis of demographic characteristics and laboratory parameters. Demographic factors included age, gender, ethnicity, poverty-to-income ratio (PIR: classified as low <1.3, middle 1.3–3.5, and high >3.5), and body mass index (BMI). Smoking and alcohol consumption data were obtained through self-reports. Existing medical conditions were assessed based on self-reported information or validated through relevant laboratory and imaging studies, including hypertension, CVD, and diabetes (DM). Laboratory parameters analyzed in this study encompassed glycated hemoglobin (HbA1c), triglycerides, total cholesterol, high-density lipoprotein (HDL), and eGFR.

### 2.5 Survival status

The NHANES data were linked with death records from the National Death Index (NDI). This study also investigated the association between OBS and all-cause mortality. Mortality data, sourced from the NDI and integrated with NHANES, were updated through December 31, 2019.

### 2.6 Statistical analysis

In this study, we followed the statistical guidelines recommended by NHANES, incorporating cycle weights into all analyses. All statistical analyses accounted for the complex, multistage sampling design of NHANES using appropriate sampling weights, strata, and primary sampling units (PSUs), as recommended by the National Center for Health Statistics. Participants were grouped based on OBS tertiles and whether they were in the advanced CKM stages. Continuous variables were reported as weighted means with standard errors, while categorical variables were presented as weighted proportions. To compare characteristics, we utilized analysis of variance (ANOVA) or the Kruskal-Wallis test for continuous variables and the chi-square test for categorical variables.

Logistic regression model was used to explore the association between OBS and advanced CKM stages. In Model 1: Unadjusted, to provide a baseline assessment. Model 2: Adjusted for covariates including sex, age, ethnicity. Model 3: Adjusted for covariates including sex, age, ethnicity, PIR, BMI, smoking status, alcohol consumption. Moreover, we also used restricted cubic spline (RCS) to examine the potential linear relationship between OBS and advanced CKM stages. With subgroup analyses, the association between OBS and advanced CKM stages was examined across age, gender, ethnicity, and PIR. To examine the association between OBS and all-cause mortality in patients with CKM, non-advanced CKM stages and advanced CKM stages, we conducted multivariate Cox regression models. In Model 1: Unadjusted, to provide a baseline assessment. Model 2: Adjusted for covariates including sex, age, ethnicity, PIR, BMI, smoking status and alcohol consumption. Model 3: Adjusted for covariates including sex, age, ethnicity, PIR, BMI, smoking status, alcohol, HbA1c, hypertension, CKD, and CVD. In addition, RCS analysis was also used to explore the potential linear association between OBS and long-term survival status.

Statistical significance was set at a two-sided *p*-value less than 0.05. All statistical analyses were conducted using R software, version 4.1.1 (R Core Team, Vienna, Austria).

## 3 Results

### 3.1 Baseline characteristics

A total of 12,793 participants were included in the final analysis dataset. The baseline characteristics of participants are presented in Table 1. The average age of the study population was 51.73 years, with 51.81% being female and 48.19% male. The overall OBS was 21.56. Advanced stages of CKM accounted for 9.8% of the total population. The overall mortality rate in this study was 5.68%, with mortality rates gradually decreasing as the OBS increased. Participants in the highest tertile (T3) of OBS were younger, more likely to be non-Hispanic White, had a higher PIR, were less likely to be current smokers or alcohol consumers, had lower BMI, glucose, HbA1c, triglyceride, and albumin, and higher eGFR compared to those in the lowest tertile (T1). We divided patients with CKM into two groups based on the presence or absence of advanced CKM stages, and conducted comparisons between the two groups. Details can be found in Supplementary Table 2.

**Table 1:** Baseline characteristics of participants according to oxidative balance score’s tertile.

Variable	Total	T1	T2	T3	*P*-value
Age, year	51.73 (0.21)	52.94(0.34)	52.00 (0.29)	50.99 (0.28)	< 0.001
Sex, %					0.63
Female	51.81	51.2	51.38	52.44	
Male	48.19	48.8	48.62	47.56	
Ethnicity					< 0.0001
Mexican American	7.54	6.9	8.35	7.11	
Non-Hispanic Black	10.09	17.51	10.91	6.22	
Non-Hispanic White	70.51	64.2	68.93	74.57	
Other Hispanic	5.22	6.06	5.28	4.81	
Other Race	6.64	5.32	6.54	7.28	
PIR					< 0.0001
Low	17.26	30.74	18.58	13.11	
Middle	32.35	40.98	36.1	30.4	
High	44.14	28.28	45.33	56.49	
Current smoking, %	18.26	32.92	19.71	10.77	< 0.0001
Current alcohol consumption, %	70.72	66.93	74.57	76.42	< 0.0001
BMI	29.58 (0.11)	30.72 (0.18)	30.20 (0.16)	28.55 (0.13)	< 0.0001
Glucose, mg/dl	108.00 (0.52)	112.89 (1.56)	109.36 (0.79)	104.71 (0.74)	< 0.0001
HbA1c	5.71 (0.01)	5.84 (0.03)	5.74 (0.02)	5.63 (0.02)	< 0.0001
Triglyceride mmol/L	1.44 (0.02)	1.52 (0.03)	1.52 (0.03)	1.35 (0.03)	< 0.0001
Total cholesterol, mmol/L	5.13 (0.02)	5.12 (0.04)	5.15 (0.03)	5.12 (0.02)	0.56
HDL, mmol/L	1.39 (0.01)	1.34 (0.01)	1.37 (0.01)	1.43 (0.01)	< 0.0001
LDL, mmol/L	3.04 (0.02)	3.06 (0.05)	3.04 (0.03)	3.02 (0.03)	0.75
Albumin, g/L	42.92 (0.06)	42.21 (0.09)	42.84 (0.08)	43.30 (0.08)	< 0.0001
eGFR	90.60 (0.30)	88.41 (0.52)	90.25 (0.39)	91.84 (0.45)	< 0.0001
CVD, %	9.02	14.49	9.6	6.19	< 0.0001
DM, %	16.18	21.76	17.64	12.53	< 0.0001
Hypertension, %	42.32	50.88	45.12	36.22	< 0.0001
CKD, %	13.52	19.31	14.31	10.53	< 0.0001
Mortality, %	5.68	9.89	5.64	3.92	< 0.0001
CKM stages					< 0.0001
Non advanced CKM stages	89.81	84.03	89.55	93.29	
Advanced CKM stages	9.8	15.97	10.45	6.71	
Total oxidative balance score	21.56 (0.13)	10.98 (0.07)	19.26 (0.05)	28.07 (0.07)	< 0.0001
Dietary oxidative balance score	17.42 (0.11)	7.73 (0.07)	15.34 (0.06)	23.35 (0.05)	< 0.0001
Lifestyle oxidative balance score	4.14 (0.03)	3.25 (0.04)	3.92 (0.04)	4.72 (0.04)	< 0.0001

### 3.2 Association between OBS and CKM stages

The associations between OBS and advanced CKM stages are shown in Table 2. In the unadjusted model (Model 1), each one-unit increase in OBS was associated with a 6% decrease in the odds of advanced CKM stages (OR 0.94, 95% CI 0.93–0.95). After adjustment for sex, age, and ethnicity (Model 2), and further adjustment for additional covariates including PIR, BMI, smoking status, and alcohol consumption (Model 3), the association remained significant, with OBS being inversely associated with advanced CKM stages (Model 3: OR 0.97, 95% CI 0.96–0.98). When analyzed by tertiles, participants in T2 and T3 had significantly lower odds of advanced CKM stages compared to those in T1 in all models. The adjusted odds ratios for T3 were 0.58 (95% CI 0.46–0.73) in Model 3.

**Table 2:** Associations between OBS and advanced CKM stages.

Variables	Model 1		Model 2		Model 3	
	OR	*P*-value	OR	*P*-value	OR	*P*-value
OBS	0.94 (0.93,0.95)	<0.0001	0.95 (0.94,0.96)	<0.0001	0.97 (0.96,0.98)	< 0.0001
Continuous variable						
Teriles						
T1	Ref		Ref		Ref	
T2	0.61 (0.51,0.74)	<0.0001	0.64 (0.52,0.79)	<0.0001	0.79 (0.63,0.99)	0.04
T3	0.38 (0.32,0.45)	<0.0001	0.43 (0.35,0.52)	<0.0001	0.58 (0.46,0.73)	< 0.01

Model 1: No adjustment.

Model 2: Adjusted for sex, age, ethnicity.

Model 3: Adjusted for covariates including sex, age, ethnicity, PIR, BMI, smoking status, alcohol consumption.

### 3.3 RCS and subgroup analysis

**Figure 1:**
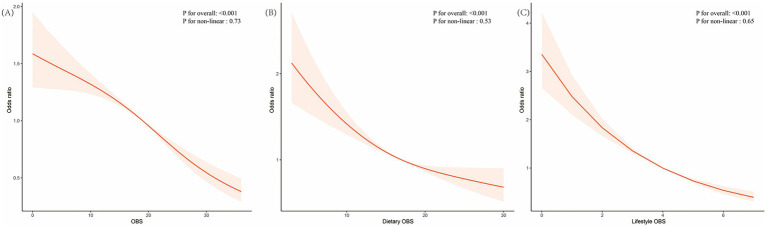
Association of OBS **(A)**, dietary oxidative balance score **(B)**, and lifestyle oxidative balance score **(C)** with advanced CKM stages evaluated by restricted cubic splines. The solid red lines represent the central estimates of the association, while the red-shaded areas denote the corresponding 95% confidence intervals. OBS, oxidative balance score.

To flexibly model the association between total OBS and risk of advanced stages of CKM, the restricted cubic spline analysis model was applied, which showed that a higher total oxidative balance score, dietary oxidative balance score, and lifestyle oxidative balance score were protective against advanced CKM stages (Figures 1A–C).

In subgroup analyses, no significant interactions were observed between OBS and any stratification factors (Supplementary Figure 2). In different age, gender, ethnicity, and PIR groups, we observed that OBS was generally negatively correlated with advanced CKM stages.

### 3.4 Correlation between OBS and all-cause mortality in all CKM stages, non-advanced CKM stages and advanced CKM stages

A median follow-up time of 80 months were obtained in all enrolled individuals. The associations of OBS with all-cause mortality across all CKM stages are detailed in Table 3. In the unadjusted model (Model 1), each one-unit increase in OBS was associated with a 6% decrease in the hazard of all-cause mortality (HR 0.94, 95% CI 0.93–0.95). This inverse association persisted after adjustment for covariates in Model 2 and Model 3, with the most significant association observed in Model 3 (HR 0.97, 95% CI 0.95–0.98).

**Table 3:** Associations of OBS with all-cause mortality in all CKM stages, non-advanced CKM stages and advanced CKM stages.

Variables	Model 1		Model 2		Model 3	
	HR	*P*-value	HR	*P*-value	HR	*P*-value
All CKM stages						
OBS	0.94 (0.93,0.95)	<0.0001	0.96 (0.95,0.97)	<0.0001	0.97 (0.95,0.98)	< 0.001
Continuous variable						
Teriles						
T1	Ref		Ref		Ref	
T2	0.54 (0.44,0.66)	<0.0001	0.57 (0.47,0.70)	<0.0001	0.69 (0.55,0.86)	< 0.001
T3	0.37 (0.28,0.48)	<0.0001	0.42 (0.32,0.55)	<0.0001	0.58 (0.42,0.78)	< 0.001
Non-advanced CKM stages						
OBS						
Continuous variable	0.95 (0.93, 0.97)	<0.0001	0.95 (0.93, 0.97)	<0.0001	0.96 (0.94, 0.99)	0.002
Teriles						
T1	Ref		Ref		Ref	
T2	0.57 (0.42, 0.78)	<0.001	0.60 (0.44, 0.81)	0.001	0.66 (0.48, 0.90)	0.01
T3	0.46 (0.32, 0.65)	<0.0001	0.49 (0.35, 0.71)	<0.0001	0.58 (0.40, 0.86)	0.01
Advanced CKM stages						
OBS						
Continuous variable	0.95 (0.94, 0.97)	<0.0001	0.95 (0.94, 0.97)	<0.0001	0.98 (0.96, 0.99)	0.01
Teriles						
T1	Ref		Ref		Ref	
T2	0.63 (0.49, 0.80)	<0.001	0.63 (0.49, 0.80)	<0.001	0.84 (0.61, 1.15)	0.27
T3	0.41 (0.30, 0.58)	<0.0001	0.41 (0.30, 0.56)	<0.001	0.62 (0.43, 0.89)	0.01

Model 1: No adjustment.

Model 2: Adjusted for covariates including sex, age, ethnicity, PIR, BMI, smoking status and alcohol consumption.

Model 3: Adjusted for covariates including sex, age, ethnicity, PIR, BMI, smoking status, alcohol, HbA1c, hypertension, CKD, and CVD.

**Figure 2:**
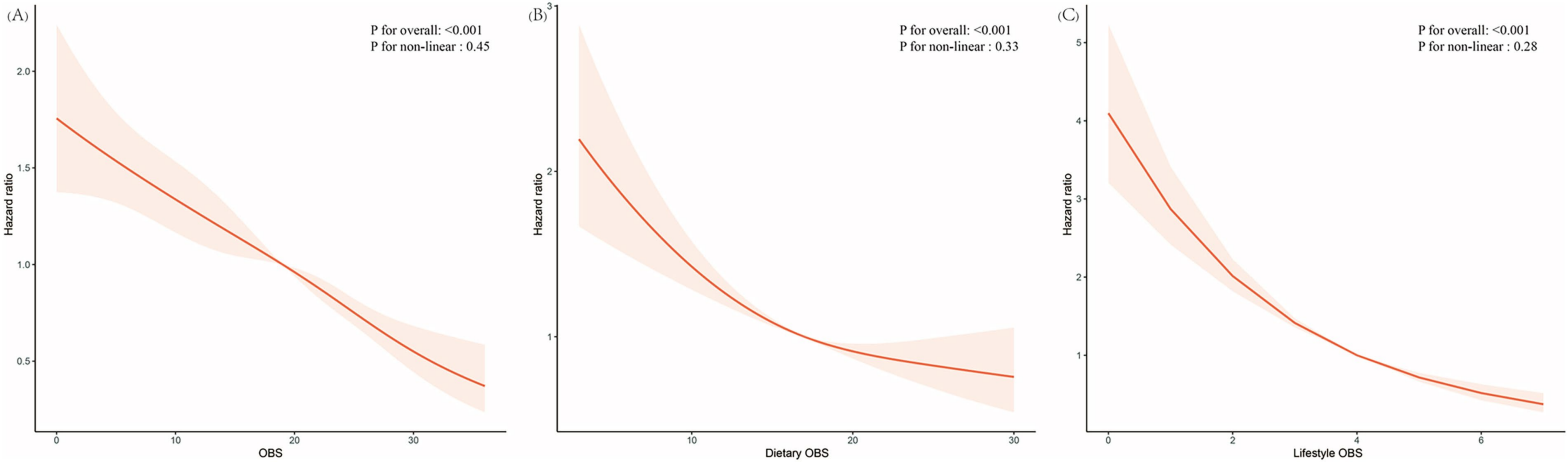
Association of OBS **(A)**, dietary oxidative balance score **(B)**, and lifestyle oxidative balance score **(C)** with all-cause mortality evaluated by restricted cubic splines. The solid red lines represent the central estimates of the association, while the red-shaded areas denote the corresponding 95% confidence intervals. OBS, oxidative balance score.

Analyzing by tertiles, participants in T2 and T3 had significantly lower hazards of all-cause mortality compared to those in T1 in all models. The adjusted hazard ratios for T3 were 0.58 (95% CI 0.42–0.78) in Model 3 for all CKM stages. In addition, RCS analysis indicated a negative relationship between total oxidative balance score, dietary oxidative balance score, and lifestyle oxidative balance score and all-cause mortality (Figures 2A–C).

In non-advanced CKM stages, the inverse association between OBS and all-cause mortality was also observed, with the adjusted hazard ratio for T3 being 0.58 (95% CI 0.40–0.86) in Model 3. In advanced CKM stages, the association was less pronounced, with the adjusted hazard ratio for T3 in Model 3 not reaching statistical significance (HR 0.62, 95% CI 0.43–0.89).

In subgroup analyses, no significant interactions were observed between OBS and any stratification factors (Supplementary Figure 3). In different age, gender, ethnicity, and PIR groups, we observed that higher OBS is associated with reduced risk of mortality.

## 4 Discussion

Our study provides compelling evidence for the protective role of oxidative balance in cardiovascular-kidney-metabolic syndrome and mortality outcomes. In a large, nationally representative sample of US adults, we found that higher oxidative balance scores were significantly associated with reduced risk of advanced CKM stages (OR 0.58, 95% CI 0.46–0.73) and lower all-cause mortality risk (HR 0.58, 95% CI 0.42–0.78). These associations demonstrated clear dose–response relationships through RCS analyses and remained consistent across various demographic and socioeconomic subgroups.

The OBS is a composite measure that assesses an individual’s oxidative stress status by evaluating both antioxidant and pro-oxidant components in diet and lifestyle (14, 15). Our study utilized a comprehensive 20-component OBS that included dietary antioxidants (fiber, β-carotene, vitamins B2, B6, B12, C, E, niacin, total folate, calcium, magnesium, zinc, copper, and selenium) and pro-oxidants (total fat, iron), as well as lifestyle factors (alcohol intake, BMI, and environmental tobacco smoke exposure assessed through plasma cotinine levels) (11). This comprehensive approach offers several advantages over single-marker measurements of oxidative stress. First, it captures the complex interplay between multiple dietary and lifestyle factors affecting oxidative balance. Second, it provides a more stable and reliable assessment of long-term oxidative stress exposure compared to individual biomarkers, which can be subject to short-term fluctuations.

The observed protective association between higher OBS and reduced risk of advanced CKM stages (42% reduction in odds) likely reflects the biological mechanisms through which oxidative stress influences disease progression. Previous studies have demonstrated that oxidative stress contributes to endothelial dysfunction, inflammation, and insulin resistance, which are key pathogenic factors in cardiovascular disease, kidney dysfunction, and metabolic disorders (25, 26). Elevated levels of ROS and oxidative stress in metabolic syndrome play a pivotal role in the pathogenesis of renal microvascular changes and both structural and functional kidney alterations (26–28). These conditions contribute to endothelial dysfunction and a prothrombotic state, which are significant in the context of CKD and CVD (26). The ROS-induced damage to the endothelium and the subsequent increase in blood clotting potential are integral to the complex pathophysiological mechanisms underlying the evolution and worsening of CKD and CVD (25). Previous clinical studies also indicated that OBS is associated with several chronic disease. In a study that included 8,095 patients with metabolic syndrome, researchers found that an increase in the OBS score was associated with a significant reduction in the prevalence of CKD (11). Similarly, it was observed that an elevated OBS score was linked to a decrease in mortality among patients with type 2 diabetes or a reduction in the prevalence of dyslipidemia and *Helicobacter pylori* infection (11, 19, 29). Our findings are consistent with previous studies, demonstrating that a high OBS score in our study significantly reduces the risk of advanced CKM. This suggests that oxidative balance may represent an independent pathway influencing CKM progression. The dose–response relationship observed in our RCS analysis further supports the biological plausibility of this association, indicating that improvements in oxidative balance may confer incremental benefits in preventing CKM progression.

Another important finding of our study was that higher OBS is associated with reduced risk of mortality in non-advanced and advanced CKM stages. The protective association between higher OBS and reduced mortality was most pronounced in individuals with non-advanced CKM stages (HR 0.58, 95% CI 0.40–0.86), while the association was somewhat attenuated in advanced stages (HR 0.62, 95% CI 0.43–0.89). This pattern suggests that the beneficial effects of maintaining optimal oxidative balance may be most effective in earlier disease stages, potentially highlighting a window of opportunity for intervention. These findings align with previous research showing that oxidative stress-related mortality risk is modified by disease severity, though our study is the first to demonstrate this specifically in the context of CKM syndrome (30–32).

Moreover, oxidative stress has been increasingly recognized as a major contributor to cerebrovascular disease, particularly stroke, which remains the second leading cause of death worldwide (33). Accumulating evidence suggests that reactive oxygen species (ROS) promote endothelial dysfunction, oxidative damage to lipids and DNA, inflammation, and blood–brain barrier disruption—key pathophysiological events leading to ischemic stroke. In patients with metabolic syndrome, who are already at elevated risk for vascular complications, an impaired oxidative balance may further exacerbate this vulnerability (34–36). Therefore, the observed inverse association between the OBS and all-cause mortality may partially reflect a reduced risk of fatal vascular events, including stroke. These findings highlight the potential of OBS as a surrogate marker for oxidative homeostasis and cerebrovascular protection. Future prospective studies are warranted to evaluate the predictive value of OBS for stroke incidence and related mortality in high-risk populations.

From a clinical and public health perspective, our findings have several important implications. The OBS represents a comprehensive measure that integrates multiple dietary and lifestyle factors affecting oxidative stress. Its strong association with both CKM progression and mortality suggests that it could serve as a valuable risk assessment tool in clinical practice. Moreover, since OBS components are largely modifiable, our results indicate that interventions targeting these factors could potentially improve outcomes in CKM patients. The consistency of these associations across different demographic groups suggests that OBS-based interventions could be broadly applicable across diverse populations.

This study has several strengths. It utilizes a large, nationally representative sample of the U.S. population, which enhances the generalizability of the findings. Additionally, we applied a multidimensional OBS that integrates both dietary and lifestyle factors, and used validated criteria for CKM staging, allowing for a robust assessment of the association between oxidative balance and cardiometabolic health. In addition, several limitations should be considered when interpreting our findings. First, the cross-sectional nature of the OBS assessment limits our ability to establish temporal relationships and causality. Second, despite comprehensive adjustment for confounders, residual confounding by unmeasured factors cannot be ruled out. Third, the OBS measurement at a single time point may not capture long-term exposure to oxidative stress. One limitation of the current study is that the Oxidative Balance Score (OBS), as originally developed, does not include polyphenols or other dietary phytochemicals, despite their well-recognized antioxidant and anti-inflammatory properties. This is largely due to the absence of standardized and comprehensive polyphenol intake data in large population-based datasets such as NHANES. Future research should address these limitations through longitudinal studies tracking changes in OBS over time and their relationship with CKM progression. Additionally, intervention studies targeting specific OBS components could help establish causality and identify the most effective strategies for improving outcomes in CKM patients.

## 5 Conclusion

In conclusion, our study demonstrates that higher oxidative balance scores are associated with significantly lower risks of advanced CKM stages and all-cause mortality in a nationally representative US population. Future research should focus on establishing causal relationships and developing targeted interventions to improve oxidative balance in patients with or at risk for CKM syndrome.

## Data Availability

Publicly available datasets were analyzed in this study. This data can be found at: https://www.cdc.gov/nchs/nhanes.
