# Supplementary material for: Association between oxidative balance score with cardiovascular - kidney - metabolic syndrome and all-cause mortality among US population
Source: Front Nutr. 2025 Aug 5;12:1564368. doi: 10.3389/fnut.2025.1564368 (PMC12360915; doi:10.3389/fnut.2025.1564368)
Supplement: Supplementary file 1 [file Table_1.docx]

Supplementary material

Supplementary table 1: Classification and Criteria for CKM Syndrome Staging

| Stage | Criteria |
| --- | --- |
| Stage 0 | - BMI: 18.5-24.9 or  - Women’s waist circumference: <88 cm  - Men’s waist circumference: <102 cm  - Excluding individuals who meet the criteria for other stages. |
| Stage 1 | - BMI ≥ 25 or  - Women’s waist circumference ≥ 88 cm  - Men’s waist circumference ≥ 102 cm or  - Prediabetes (fasting blood glucose: 100-124 mg/dL, HbA1c: 5.7%-6.4%, or on diabetes medication) |
| Stage 2 | - Metabolic risk factors: fasting serum triglycerides ≥ 135 mg/dL, hypertension, diabetes, or metabolic syndrome or  - CKD: moderate to high risk according to KDIGO guidelines. |
| Stage 3 | - CKD: very high risk according to KDIGO guidelines or  - 10-year cardiovascular disease risk ≥ 20% (Calculated by AHA PREVENT equation). |
| Stage 4 | - A history of cardiovascular disease (e.g., coronary artery disease, angina, myocardial infarction, heart failure, or stroke). |

Supplementary table 2 Baseline characteristics between non advanced CKM stages and advanced CKM stages

| Variable | Total | Non advanced CKM stages | Advanced CKM stages | P-value |
| --- | --- | --- | --- | --- |
| Age | 51.71(0.21) | 50.55(0.22) | 62.35(0.34) | < 0.0001 |
| Sex |  |  |  | < 0.001 |
| Female | 51.82 | 52.78 | 43.05 |  |
| Male | 48.18 | 47.22 | 56.95 |  |
| Ethnicity |  |  |  | < 0.0001 |
| Mexican American | 7.55 | 7.84 | 4.88 |  |
| Non-Hispanic Black | 10.08 | 9.79 | 12.74 |  |
| Non-Hispanic White | 70.5 | 70.33 | 72.02 |  |
| Other Hispanic | 5.23 | 5.38 | 3.84 |  |
| Other Race | 6.65 | 6.66 | 6.52 |  |
| PIR |  |  |  | < 0.0001 |
| Low | 17.25 | 17.25 | 29.06 |  |
| Middle | 32.36 | 33.97 | 39.55 |  |
| High | 44.13 | 48.78 | 31.39 |  |
| Current smoking status | 18.24 | 17.32 | 26.6 | < 0.0001 |
| Current alcohol consumption | 70.79 | 75.44 | 61.12 | < 0.0001 |
| BMI | 29.56(0.11) | 29.32(0.11) | 31.73(0.27) | < 0.0001 |
| Glucose, mg/dL | 107.98(0.52) | 106.20(0.54) | 122.62(2.13) | < 0.0001 |
| HbA1c | 5.71(0.01) | 5.66(0.01) | 6.23(0.04) | < 0.0001 |
| Triglyceride, mmol/L | 1.44(0.02) | 1.42(0.02) | 1.63(0.06) | 0.001 |
| Total cholesterol, mmol/L | 5.13(0.02) | 5.18(0.02) | 4.71(0.04) | < 0.0001 |
| HDL, mmol/L | 1.39(0.01) | 1.41(0.01) | 1.28(0.01) | < 0.0001 |
| LDL, mmol/L | 3.04(0.02) | 3.09(0.02) | 2.61(0.05) | < 0.0001 |
| Albumin, g/L | 42.93(0.06) | 43.07(0.06) | 41.70(0.10) | < 0.0001 |
| eGFR | 90.63(0.30) | 92.39(0.33) | 74.49(0.78) | < 0.0001 |
| DM, % | 16.13 | 13.46 | 40.6 | < 0.0001 |
| Hypertension, % | 42.27 | 38.77 | 74.39 | < 0.0001 |
| CKD, % | 13.48 | 10.76 | 38.94 | < 0.0001 |
| Mortality, % | 5.62 | 4.15 | 19.13 | < 0.0001 |
| Total oxidative balance score | 21.57(0.13) | 21.86(0.13) | 18.98(0.25) | < 0.0001 |
| Dietary oxidative balance score | 17.43(0.11) | 17.64(0.11) | 15.47(0.22) | < 0.0001 |
| Lifestyle oxidative balance score | 4.15(0.03) | 4.21(0.03) | 3.51(0.06) | < 0.0001 |


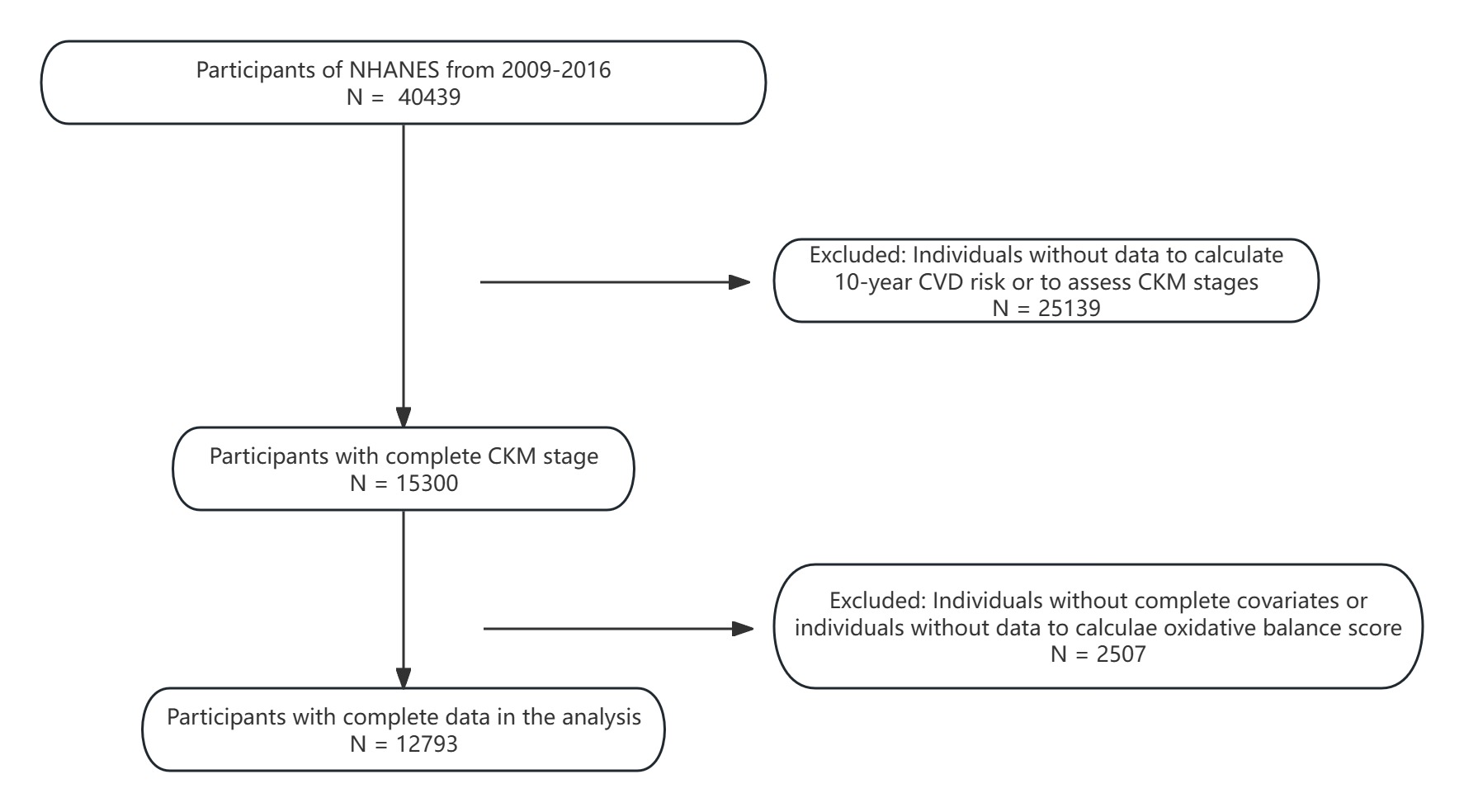


Figure 1: The flow-chart of our study


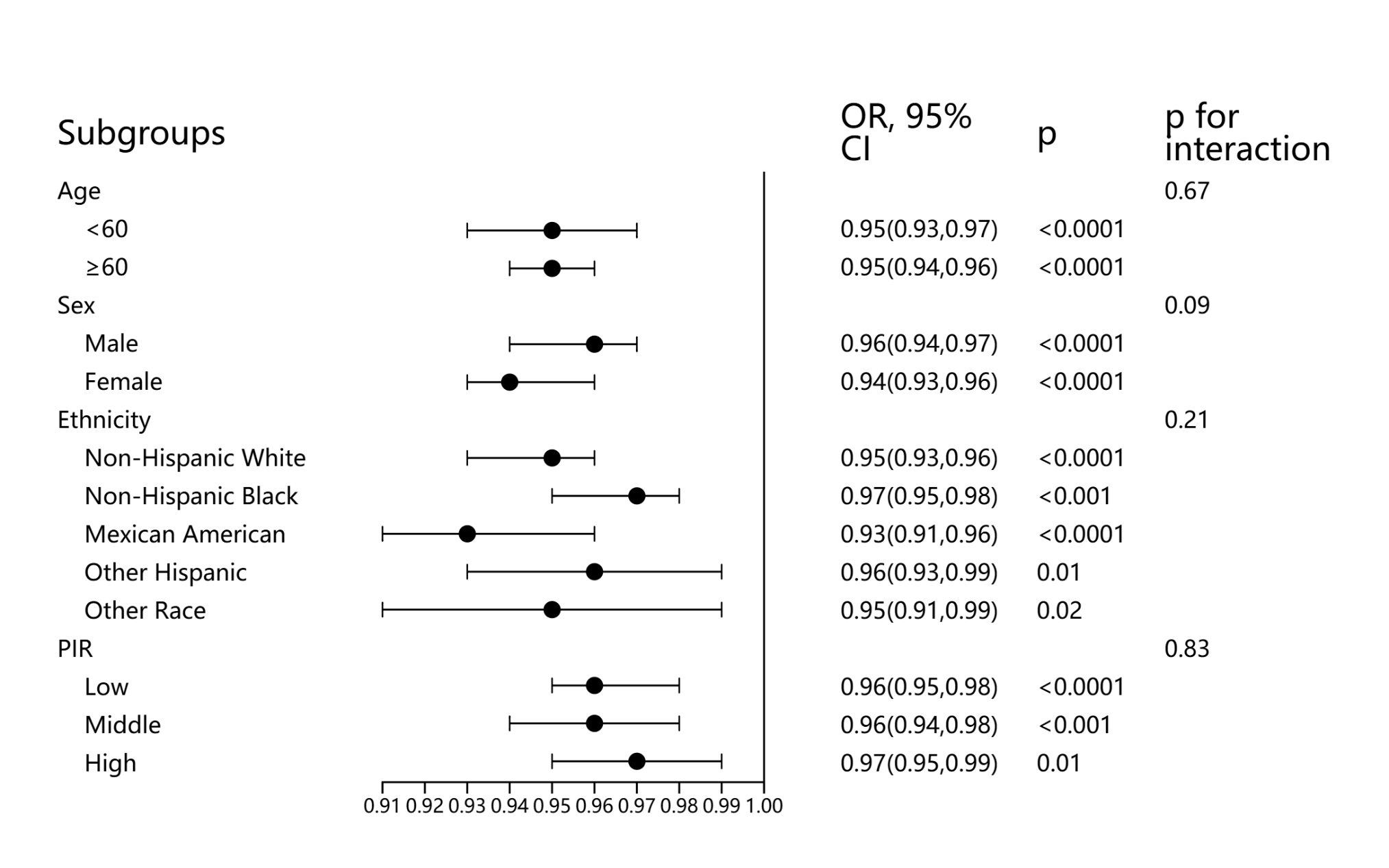


Figure 2: Subgroup analysis: association between OBS and advanced CKM stages across various groups.


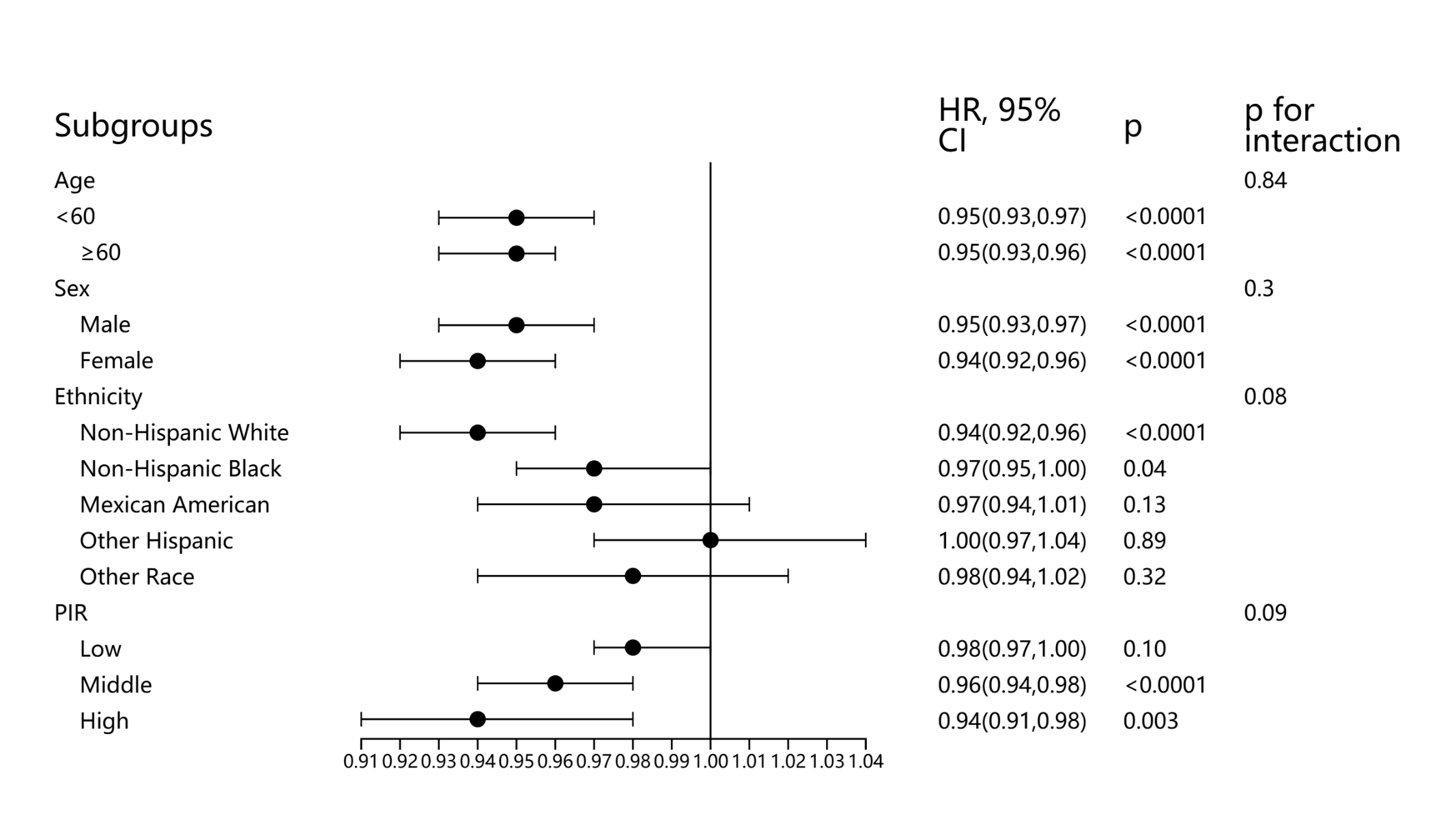


Figure 3: Subgroup analysis: association between OBS and all-cause mortality across various groups.
